# Mass azithromycin distribution and nutritional status among children aged 1–59 months: A secondary analysis of a cluster randomized trial

**DOI:** 10.1371/journal.pgph.0007473

**Published:** 2026-10-13

**Authors:** Mamadou Ouattara, Huiyu Hu, Mamadou Bountogo, Boubacar Coulibaly, Valentin Boudo, Guillaume Compaoré, Clarisse Dah, Elodie Lebas, Kieran S. O’Brien, Benjamin F. Arnold, Thomas M. Lietman, Ali Sié, Catherine E. Oldenburg

**Affiliations:** 1 Centre de Recherche en Sante de Nouna, Nouna, Burkina Faso; 2 Francis I Proctor Foundation, University of California, San Francisco, California, United States of America; 3 Department of Ophthalmology, University of California, San Francisco, California, United States of America; 4 Department of Epidemiology & Biostatistics, University of California, San Francisco, California, United States of America; ICMR-National Institute for Research in Tuberculosis: National Institute of Research in Tuberculosis, INDIA

## Abstract

Twice yearly distribution of azithromycin has been shown to reduce all-cause child mortality in several contexts. Azithromycin likely reduces mortality via reduction in clinical or subclinical infections. Treating infections may improve child nutritional status to further reduce mortality risk. Here, we used data from a placebo-controlled cluster randomized trial of azithromycin for prevention of child mortality in Burkina Faso to evaluate the effects of twice-yearly azithromycin on mid-upper arm circumference (MUAC) in children aged 1–59 months and weight-for-age Z-score (WAZ) in infants aged 1–11 months. The Child Health with Azithromycin Trial (CHAT) was a 1:1 cluster randomized trial of twice-yearly azithromycin compared to matching placebo for prevention of all-cause mortality in children aged 1–59 months. Measurements were collected every 6 months, including MUAC measurements in all children and weight measurements in infants aged 1–11 months to calculate weight-based dosing. We evaluated MUAC and WAZ at the community level and longitudinally at the individual child level, accounting for the cluster randomized design of the trial. 80,282 children aged 1–59 months, of whom 26,983 were infants aged 1–11 months, in 284 communities were included in analyses. At the community level, we found no evidence of a difference in mean MUAC or WAZ after 36 months or over time. There was no evidence of a difference in change over time in MUAC (*P*-value for interaction = 0.94) or WAZ (*P* = 0.18) at the individual level. We found no evidence to support an effect of twice-yearly mass azithromycin distribution on measures of undernutrition in children in this setting in Burkina Faso.

**Trial registration:**

ClinicalTrials.gov NCT03676764

## Introduction

Twice yearly mass distribution of azithromycin has been shown to reduce all-cause child mortality in children aged 1–59 months in high mortality settings [1–3]. Azithromycin is a broad-spectrum antibiotic, and this effect may in part be due to reductions in transmission of pathogens. While the mechanisms underlying mortality reductions with azithromycin are incompletely understood, previous work has suggested that azithromycin reduces mortality due to a number of infectious causes [4]. Some studies have shown that antibiotic use increases child growth in children with pre-existing morbidity or infection [5,6]. Individually randomized trials of azithromycin in infants without pre-existing morbidity have not shown evidence of an effect of azithromycin compared to placebo on growth endpoints [7,8]. Similarly, individual distribution of azithromycin to infants has not been shown to reduce mortality [9,10]. There may be effects at the community level that are not observed with individual dosing [9,10].

Studies of mass distribution of azithromycin for child survival and trachoma have not found evidence of an effect of azithromycin for child nutritional status [11–14]. These studies have generally evaluated nutritional status at a single time point or longitudinally several years apart. In the present study, we used data from a cluster randomized trial in Burkina Faso [1] that collected mid-upper arm circumference (MUAC) measurements on all children and weight measurements on all infants <12 months of age to evaluate the impact of twice-yearly distribution of azithromycin versus placebo on both point prevalence of undernutrition and longitudinal growth in children with repeated anthropometric measurements collected every 6 months. We hypothesized that anthropometric indicators of nutritional status would be higher in communities receiving twice-yearly azithromycin compared to placebo.

## Methods

### Ethics statement

The trial was reviewed and approved by the Institutional Review Board at the University of California, San Francisco and the Comité National d’Ethique pour la Recherche in Ouagadougou, Burkina Faso. Written informed consent was obtained from the caregiver of each participant.

### Trial methods

The Child Health with Azithromycin Treatment (CHAT) trial was a cluster randomized placebo-controlled trial evaluating the effect of twice-yearly mass azithromycin distribution compared to placebo for prevention of child mortality [1,15]. The study was conducted from August 2019 through February 2023. The primary outcome, all-cause mortality among children aged 1–59 months, has been previously reported [1]. Here, we report anthropometric outcomes that were not pre-specified in the trial’s analysis plan.

### Study setting

The CHAT trial was conducted in Nouna District in northwestern Burkina Faso. The district is rural and largely agrarian, with seasonal rainfall from June through September and an annual harvest in November [16]. Undernutrition is common in Nouna, with a wasting prevalence (weight-for-height Z-score, WHZ, < -2) reported at 6% and stunting prevalence (height-for-age Z-score, HAZ) of 24% in 2017–2019 [17]. All communities in the district were eligible to participate, however an escalating insecurity situation with increased risk of kidnapping and terrorist attacks over the course of the trial meant that some communities were no longer safe to access by the study team. Communities that were inaccessible were eligible to re-enter the study if it became safe for them to do so.

### Communities and participants

Prior to the baseline study census in the parent trial, a census of all communities and structures in the district was undertaken. All communities that agreed to participate in the study were eligible for participation and were randomized, with the exception of the town of Nouna, which was excluded because it is more urban and has lower mortality rates. Communities with a census population of 2000 or more were split into multiple randomization units (“clusters”), so each cluster had a population of <2000 individuals. During the study, a census was conducted twice yearly to enumerate all children in the study area. Study treatment was provided during the census. Children were eligible for treatment if they were between 1 and 59 months of age, had no documented allergies to macrolides, and the caregiver consented to their participation in the study.

### Randomization, interventions, and masking

Communities were randomized in a 1:1 fashion to twice-yearly azithromycin distribution or placebo to children aged 1–59 months. Communities in the azithromycin group received a single, oral 20 mg/kg dose of azithromycin delivered to all children aged 1–59 months, with the dosing based on weight for children < 12 months of age or based on height stick approximation for children ≥ 12 months [18,19]. Communities in the placebo arm received an equivalent volume of matching placebo. The placebo was identical to the azithromycin in taste and appearance to facilitate masking. Families, study staff, and investigators were masked to treatment allocation.

### Anthropometric measurements

Weight measurements were taken among infants < 12 months of age to facilitate dosing at each biannual census prior to treatment administration. MUAC measurements were collected on all children regardless of age. Children with MUAC measurements < 11.5 cm were referred to care for severe acute malnutrition (SAM) care and were retained in the analysis.

### Statistical analysis

This was a non-prespecified secondary analysis of the CHAT trial [1]. The sample size for CHAT was based on the primary outcome, all-cause child mortality, with a fixed sample size of 341 clusters based on the number of clusters identified during study planning. Community-level means for MUAC and WAZ were used to assess the impact of azithromycin at the community level. Children were linked with a unique identification number across multiple time points as part of the census conducted every 6 months. These longitudinal measurements were used to assess changes in MUAC over time at the individual level in children and 6-month changes in WAZ in the subset of infants with two measurements. All analyses were intention-to-treat and were conducted as complete-case analyses.

### Community level analysis

We summarized mean MUAC and WAZ at each census round by cluster, then aggregated these to estimate arm-specific means and 95% confidence intervals over time. We also plotted the mean MUAC and WAZ trajectories across study phases by treatment arm to visualize temporal trends. In addition, linear mixed-effects models were fitted for mean cluster-level MUAC and WAZ, including treatment arm, census round, and treatment arm by census round interaction term, with random intercepts for clusters to account for repeated measures. As a non-prespecified sensitivity analysis, we assessed whether azithromycin improved outcomes in children with low MUAC (<12.5 cm) and infants with low WAZ (<-2). For each model, a treatment by baseline nutritional status interaction term was added to the model to assess the interaction between azithromycin vs placebo and baseline nutritional status. As a randomized controlled trial, there was no confounding by definition in these analyses and there was no adjustment for confounding. Exploratory subgroup analyses were based on characteristics for which we hypothesized there could be potential differences in the efficacy of azithromycin (e.g., age, baseline nutritional status).

### Individual-level analysis

We fitted linear mixed-effects models to account for repeated measurements within children and clustering by community. For MUAC, the model included treatment arm, census round, child age at visit, and baseline MUAC, with random intercepts for children nested within clusters to account for the longitudinal structure of the data. For WAZ, which is inherently age-standardized, models were fitted with treatment arm and baseline WAZ as covariates. Because weight was only measured for children aged 1–11 months, resulting in two timepoints, we included random intercepts only at the cluster level. Baseline age was restricted to children younger than 6 months. As a non-prespecified sensitivity analysis, we further explored potential effect modification by age on both MUAC and WAZ outcomes by including an age-by-treatment interaction term. Age was modeled as a continuous variable (age in months), included both as a linear covariate and with an age by treatment arm interaction term. The interaction was assessed using a likelihood ratio test comparing the model with versus without the interaction term. All analyses were conducted in R (version 4.4).

## Results

Of 341 communities randomized to twice yearly azithromycin or placebo, 284 entered into the study during the first census (N = 144 azithromycin, N = 140 placebo, Fig 1). A total of 80,282 children (N = 40,197 azithromycin, N = 40,085 placebo) and 26,983 infants (N = 13,763 azithromycin, N = 13,220 placebo) were included in community-level analyses for MUAC and WAZ outcomes, respectively. A total of 64,762 children (N = 32,706 azithromycin, N = 32,056 placebo) had more than one 6-monthly MUAC measurement and 10,271 infants (N = 5,251 azithromycin, N = 5,020 placebo) had more than one 6-monthly WAZ measurement and contributed to individual-level analyses. Baseline characteristics were similar in azithromycin and placebo-treated children (Table 1).

**Table 1:** Baseline demographic characteristics.

	Azithromycin	Placebo
Number of communities	144	140
Number of children (1–11 months) with WAZ	3,206	3,204
Number of children (1–59 months) with MUAC	19,986	20,261
Child’s sex among children aged 1–11 months^1^		
Female	1,516 (47.3%)	1,598 (49.9%)
Male	1,690 (52.7%)	1,606 (50.1%)
Child’s sex among children aged 1–59 months^1^		
Female	9,837 (49.2%)	10,059 (49.6%)
Male	10,149 (50.8%)	10,202 (50.4%)
Age among children aged 1–11 months, month^2^	6 (3–8)	6 (3–8)
Age among children aged 1–59 months, month^2^	34 (20–47)	34 (20–47)
WAZ^2^	-0.92 (-1.86 to -0.01)	-0.9 (-1.9 to 0.03)
MUAC, cm^2^	14.6 (14.0 to 15.5)	14.5 (13.8 to 15.3)

Abbreviations: WAZ, Weight-for-Age z-score; MUAC, Mid-Upper Arm Circumference; IQR, interquartile range**;** ^1^Numbers presented as N (%); ^2^Numbers presented as median (IQR).

**Fig 1:**
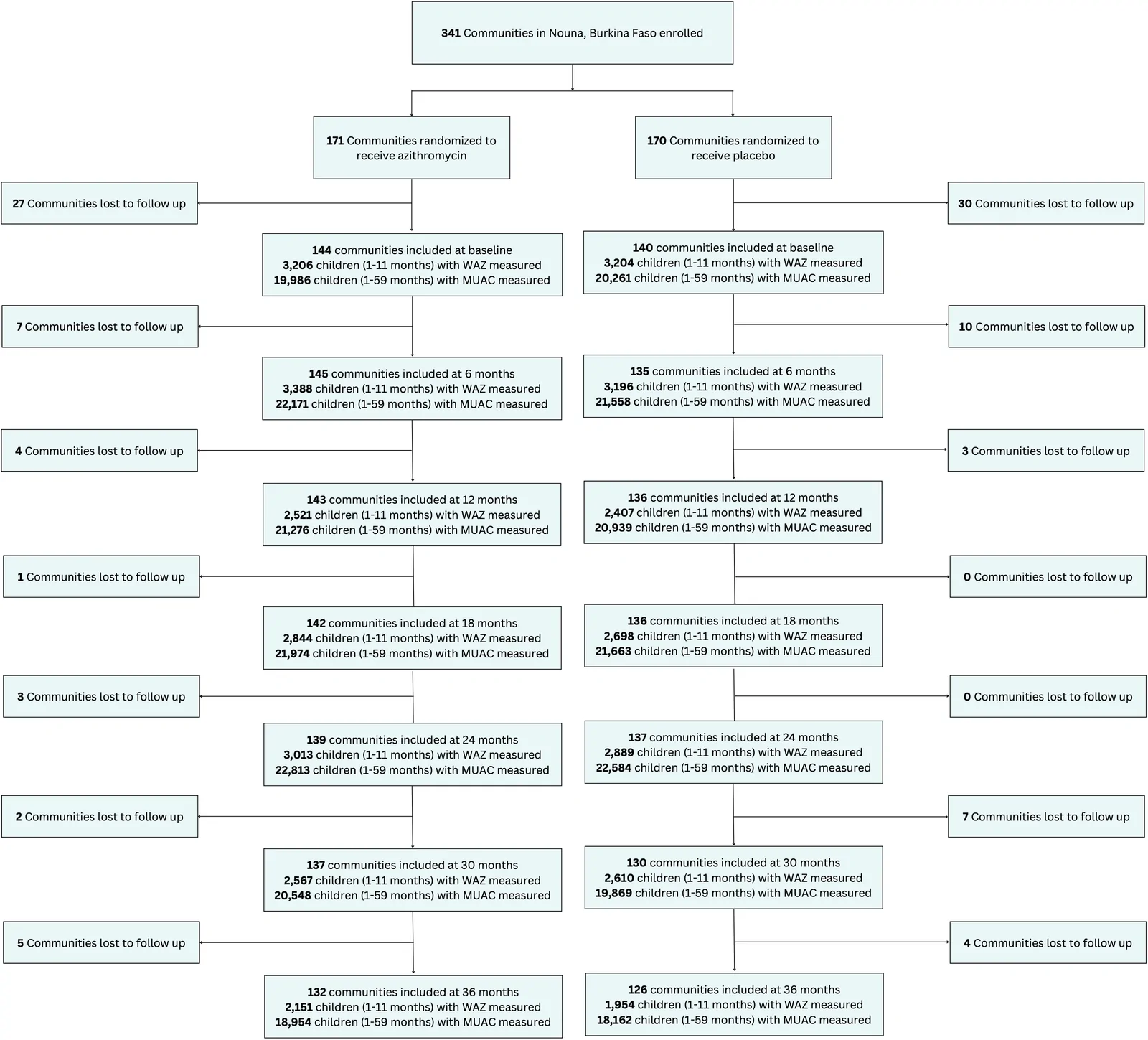
CONSORT flow diagram of included communities and participants.

At the community level, we found no evidence of a difference in mean MUAC or WAZ 36 months or at any interim time point (Fig 2). At 36 months, mean MUAC was 14.7 cm in each, with a difference of -0.01 cm (95% CI: -0.13 to 0.11; p = 0.90). The mean WAZ at 36 months was -0.81 in the azithromycin group and -0.95 in the placebo group, yielding a difference of -0.14 (95% CI: -0.41 to 0.13; p = 0.30). There was no evidence of a difference in MUAC or WAZ over time (overall treatment arm by time P-value = 0.18 for MUAC and 0.19 for WAZ). In subgroups of children defined by low MUAC (MUAC < 12.5 cm) and infants with low WAZ (WAZ < -2) there was no evidence of a difference in the effect of azithromycin vs placebo compared to those without low MUAC or low WAZ. A total of 1,874 children in the azithromycin group and 2,014 children in the placebo group had low MUAC. The stratum-specific effect of azithromycin in children with low MUAC was consistent with a 0.004 cm difference (95% CI -0.07 to 0.08 cm, P = 0.92, P for interaction 0.16). The stratum-specific effect of azithromycin in infants with low WAZ was consistent with a mean difference of 0.1 SD (95% CI -0.08 to 0.3, P = 0.29, P for interaction 0.80).

**Fig 2:**
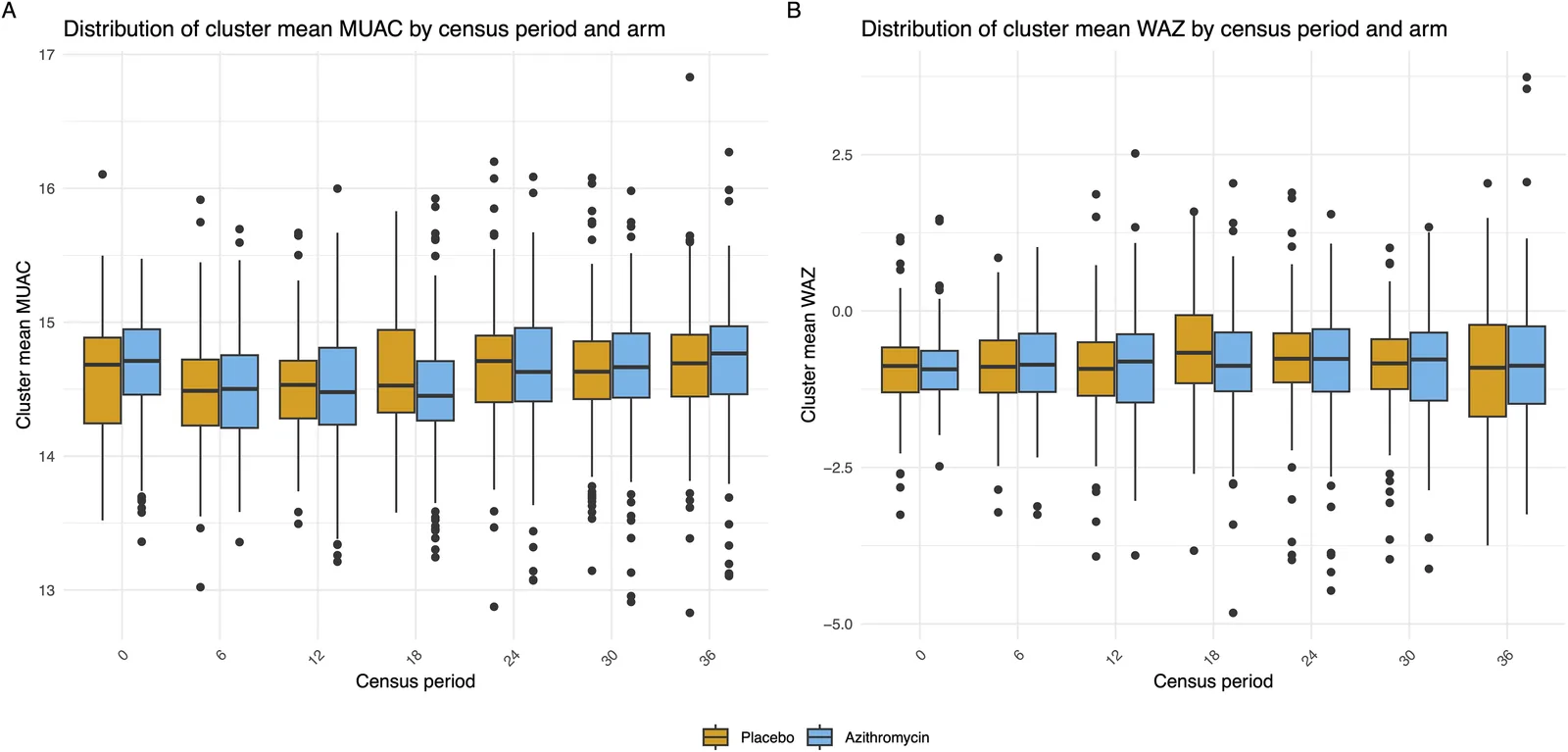
Anthropometric measurements by treatment group over time at the cluster level. **A.** Mean mid-upper arm circumference (MUAC) in children aged 1-59 months. **B.** Weight-for-age Z-score (WAZ) in children aged 1-11 months.

At the individual level, MUAC increased over time in both groups but there was no evidence of a difference in MUAC by group over time (P-value for interaction = 0.94; Fig 3). Overall, younger children were more likely to have increased MUAC if they were in the azithromycin arm compared to placebo versus older children (*P*-value for interaction < 0.001). The 95% confidence interval included 0 (no effect) for the difference in MUAC in azithromycin compared to placebo communities across all age groups (S1 Fig), suggesting no strong evidence of an effect in any given age group. In analyses of WAZ in infants with two WAZ measurements (i.e., infants < 6 months at the first measurement), WAZ was -1.1 (95% CI -1.2 to -1) in infants in azithromycin communities compared to -1.2 in placebo communities (95% CI -1.3 to -1.1). This corresponded to a mean difference of 0.08 (95% CI -0.04 to 0.21, *P* = 0.18) in azithromycin compared to placebo communities (Fig 3). The effect of azithromycin compared to placebo increased with age among infants aged 1–5 months at their first measurement, but the P-value for interaction was not statistically significant (*P* = 0.12; S1 Fig).

**Fig 3:**
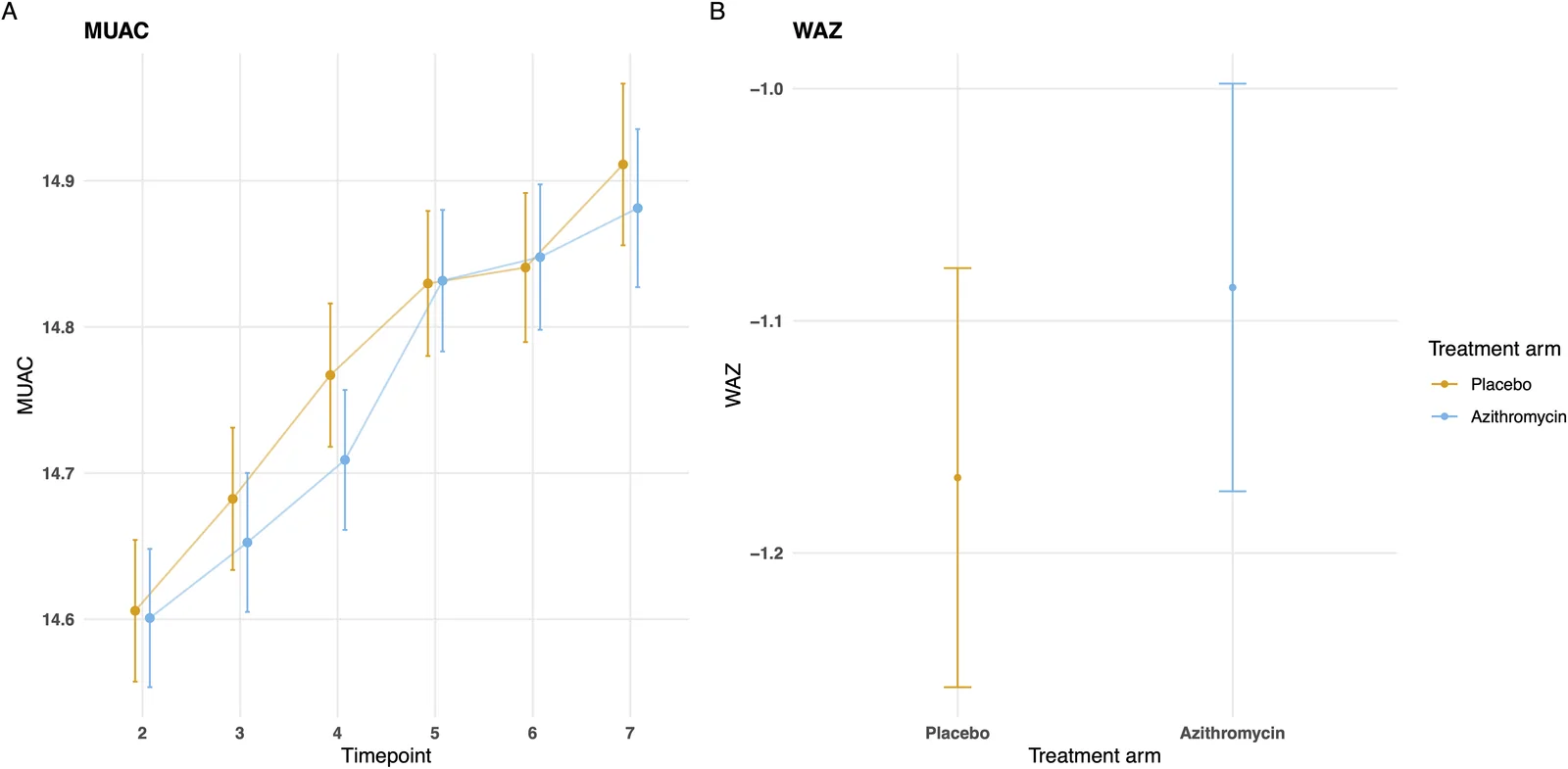
Individual-level analyses of azithromycin compared to placebo. **A.** Mid-upper arm circumference (MUAC) over time in children aged 1-59 months with >1 MUAC measurement; **B.** Weight-for-age Z-score (WAZ) in infants age 1-5 months at the first measurement with one additional WAZ measurement. Panel B depicts mean WAZ with 95% confidence intervals at the second measurement (6 months later) in the azithromycin compared to placebo group. For both panels, yellow indicates the placebo and blue indicates azithromycin group.

## Discussion

In this non-prespecified secondary analysis, we found no evidence to support an effect of twice-yearly azithromycin distribution on anthropometric measurements at the community or individual level after 36 months of distributions. Although we hypothesized that mass azithromycin distributions may improve nutritional status by reducing clinical or subclinical infections (e.g., due to enteric infections) [4,20], there was no evidence of an effect on MUAC in children under age 5 or WAZ in infants under 12 months of age. These results are consistent with previous studies which have failed to show an effect of individual or mass distribution of azithromycin on child growth [5,7,8,12–14,21]. In contrast, a meta-analysis of trials of antibiotic use in children with morbidity (e.g., HIV or malnutrition) suggested that antibiotics may be growth promoting in these children [5]. The results of this meta-analysis were largely driven by a trial of antibiotics for uncomplicated severe acute malnutrition [22]. Antibiotics are included as part of routine treatment for uncomplicated severe acute malnutrition for children without clinical signs of infection due to the potential for asymptomatic infection in these children. In general, antibiotic trials in this population have shown short-term (up to 8 weeks) but not longer-term effects on weight gain [23,24]. It is possible that azithromycin has a similar short-term effect in well-nourished children that was not captured in the present study due to the timing of measurements. However, these results suggest that growth promotion may not be a major contributing mechanism for the child survival benefits of mass azithromycin distribution.

The age structure of this population of children under age 5 skewed slightly older, with a median age of 34 months. Younger children have the highest risk of mortality and the highest risk of malnutrition [25]. Previous trials of azithromycin for prevention of infant mortality have shown no effect of azithromycin on anthropometric outcomes at 6 months of age [7,8]. These studies evaluated individual dosing of azithromycin. Studies of individual (rather than community-wide) distribution of azithromycin have shown no evidence of a benefit of azithromycin for prevention of mortality [9,10]. In contrast, mass distribution of azithromycin to entire populations of children under age 5 have consistently shown a preventive effect of azithromycin for child mortality [1–3]. However, this effect was largely attenuated when only infants were treated, suggesting that treatment of all children is necessary to see measurable effects on mortality [2]. In this study, there was some suggestion of differential effects by age, but these analyses were exploratory and the study was underpowered for age-specific subgroup analyses. These results may not generalize to populations with different age structures, and future studies evaluating effects of azithromycin on anthropometric outcomes in younger children are warranted.

Because these data were collected as part of a large, simple cluster randomized controlled trial, data collection was limited to MUAC in all children and weight in infants due to logistics and feasibility in transporting anthropometric equipment to the field. As a result, height was not collected and thus we were unable to evaluate the impact of azithromycin in linear growth. Stunting, or a low height-for-age Z-score (HAZ), reflects chronic undernutrition and is an important indicator for monitoring malnutrition. Antibiotic trials have generally shown increased impact of antibiotics on weight gain rather than linear growth [5], perhaps due to the antibiotic’s shorter-term but not longer-term effects. Whether repeated dosing at regular intervals with an antibiotic, such as in mass drug administration programs for prevention of mortality that distribute antibiotics every 6 months and may occur for several years of a child’s life, affects linear growth differently than individual intermittent dosing for specific episodes of illness is unknown. Children in this study were relatively well nourished. Given the study’s sampling (all children in the study communities based on a door-to-door census), this reflects a well-nourished population. These results may not be generalizable to populations with a higher prevalence of underweight or wasting. The parent study found a relatively large effect of azithromycin for prevention of mortality in the same population, although there was some signal that effects on mortality were greater in the malnourished children [1,26]. Despite an overall mortality benefit in this subgroup, we found no evidence of a difference in MUAC or WAZ in children with low MUAC or infants with underweight at baseline. It is possible these results may not generalize to populations with different etiology of malnutrition.

The results of this study must be interpreted in the context of several limitations. As previously mentioned, measurements were collected at 6-month intervals that would not capture any effect of antibiotics on shorter-term growth that did not persist to 6 months. Weight measurements were only collected in children < 12 months of age, as they were collected as part of weight-based dosing for infants. These measurements were collected for the purposes of calculating the dose of azithromycin or placebo using hanging infant scales, which may have introduced measurement error. We anticipate that any measurement error in weight measurements for infants would be non-differential with respect to azithromycin or placebo, due to the masked nature of the trial, and thus would attenuate effects towards the null. This study evaluated twice-yearly distribution of single-dose azithromycin in the general population of children aged 1–59 months. While there is some evidence that the effects of azithromycin for mortality are stronger in children with undernutrition [26–28], current evidence does not support targeted azithromycin distribution for mortality. Whether different dosing strategies, timing, or duration would yield different effects is not known. While we hypothesized that azithromycin could impact growth via reductions in infection, we do not have infection data cause-specific mortality results, which suggested a reduction in infectious mortality in the parent trial [29]. Finally, the results of this study can likely only be generalized to settings with a similar distribution of age, malnutrition, food insecurity, dietary practices, and background antibiotic use. While the study sample is representative of this population in rural Burkina Faso, it may not be representative of other areas of West Africa or other geographic regions where azithromycin may be considered as an intervention to prevent child mortality.

Overall, we found no evidence of an effect of twice-yearly azithromycin distribution on MUAC in children aged 1–59 months or WAZ in infants aged 1–11 months 6 months after distribution. Changes in ponderal growth may not be a major contributor to the protective effect of azithromycin for child mortality in Burkina Faso.

## Supporting information

S1 Fig Estimated treatment effect of azithromycin versus placebo on MUAC (A) and WAZ (B) by child’s age in months. Panel A shows the estimated difference in MUAC in CM with points for point estimates and shaded area representing 95% confidence intervals. Panel B shows estimated difference in WAZ with dots as point estimates and bars for 95% confidence intervals.
(TIFF)

S1 Protocol Protocol for the parent trial. (DOC)

S1 Text Statistical analysis plan for the parent trial. (PDF)
