# Supplementary material for: Mass azithromycin distribution and nutritional status among children aged 1–59 months: A secondary analysis of a cluster randomized trial
Source: PLOS Glob Public Health. 2026 Oct 13;6(10):e0007473. doi: 10.1371/journal.pgph.0007473 (PMC13660883; doi:10.1371/journal.pgph.0007473)
Supplement: Supplementary file 1 [file pgph.0007473.s003.pdf]

CHAT:
A community-randomized trial

February 13, 2023

Version 1.6

Statistical Analysis Plan

UCSF Francis I. Proctor Foundation
Centre de Recherche en Sante de Nouna
University of Heidelberg

Introduction

This document (Statistical Analysis Plan, SAP) describes the planned analysis and reporting for the clinical trial, **Azithromycin for the prevention of post-neotanal childhood mortality in Burkina Faso: a community-randomized trial**. It includes specifications for the statistical analyses and tables to be prepared for the interim and final Clinical Study Report. This study is designed to compare methods to reduce childhood mortality using mass administration of azithromycin (Pfizer, CAS 83905-01-5) compared to placebo. The content of this Statistical Analysis Plan meets the requirements stated by the US Food and Drug Administration and conforms to the American Statistical Association's Ethical Guidelines.

The following documents were reviewed in preparation of this Statistical Analysis Plan:

- Azithromycin for the prevention of infant and child mortality in Burkina Faso: a community-randomized trial, Manual of Operations
- Azithromycin for the Prevention of Neonatal, Infant, and Child Mortality in Burkina Faso and Safety Assessment, Proposal
- Statistical Analysis Plan, MORDOR Trial
- ICH Guidance on Statistical Principles for Clinical Trials

The planned analyses described in this SAP will be included in future manuscripts. Exploratory analyses not necessarily identified in this Statistical Analysis Plan may be performed to support the analysis. Unplanned analyses not delineated in this Statistical Analysis Plan will be documented as such in the final Clinical Study Report and manuscripts.

This document will be reviewed prior to the enrollment of patients. All subsequent changes will be indicated by detailed change log in the Appendix.

Contents

1 Summary **4**
1.1 Mortality Trial 4

2 Statistical Analysis **4**
2.1 Planned Analyses 4
2.1.1 Mortality Trial 4
2.2 Interim Monitoring 7

3 Sample Size Considerations **8**

4 Randomization **10**

5 Abbreviations **11**

6 Revision History **12**

1 Summary

1.1 Mortality Trial

The trial profile is given in the Manual of Operations. In the mortality trial, communities are randomized to two arms: **Azithro** and **Placebo**. The trial is a placebo-controlled, double-masked (i.e., double-blind), community-randomized, clinical trial.

Objective. Determine the efficacy of biannual mass azithromycin distribution versus placebo in children aged 1–59 months on all-cause mortality. *We hypothesize that biannual distribution of azithromycin will lead to significantly reduced all-cause mortality among children aged 1–59 months after 36 months of treatment.*

2 Statistical Analysis

2.1 Planned Analyses

2.1.1 Mortality Trial

The primary prespecified analysis will use the number of deaths in each community as the outcome variable. For analysis, a death is counted when an individual in the target age range 1–59 months is enumerated as alive on census i ($i = 0, 1, 2, 3$) and is not on census $i + 1$ due to death. Census i takes place at the beginning of the study, and each subsequent census takes place approximately six months later.

Primary Analysis.

The primary analysis will be conducted as **negative binomial regression**. The number of person-years (for persons in the target age range) is derived by summing the person-times for each period. The community-level predictor consists of treatment arm for the community. Specifically, the treatment variable is a community level variable, not an individual level variable. The statistical significance assessed by permutation testing at the level of the randomization unit.

Individuals who are in the target age range at the beginning of any study period, but who are no longer in the target age range by the end, are considered to be fully enrolled and contribute the full amount of person-time for that period.

The analysis will be **two-sided**, with a **type I error rate** (alpha) of 0.05.

Statistical considerations.

- Statistical tests will be conducted with Monte Carlo permutation based on the randomization unit. The number of replications will be 10,000, unless the Monte Carlo confidence interval for the P-value includes 0.05. In this case, 10,000,000 will be used, and this fact reported.

- Tests for interaction by age sub-groups and by phase on the additive scale will be conducted to evaluate for statistically significant subgroup differences.

- Model adequacy will be checked by examination of residuals or other goodness of fit tests as needed. Inadequate model fit will prompt us to report alternative models.

- For negative binomial regression, an aggregation parameter of 30 or greater will cause us to re-fit using Poisson regression.

- Multiple imputation will be used in case of missing baseline covariates (if applicable). Missing outcome variables will be handled by sensitivity analysis and reporting of conditional results.

- Individual level missing data (due to loss to follow-up or dropouts) is expected. If an individual is lost to follow-up or otherwise unavailable during an interval of length D, then the individual will not be assigned any amount of person time. If an individual dies within an interval of length D, then the individual is assumed to contribute the amount of person-time $D/2$ to the denominator (midpoint imputation).

- Village-level missing data could occur for various reasons, including field error, withdrawal of support from village leadership, or other unforseen reasons. The analytic

plan will be to analyze all available information (including data from any community prior to the time of dropout of that community). If a community drops out and then re-enters, all available data (before and after the dropout period) will be analyzed.

Supplementary analyses.

The purpose of the supplementary analyses reported in this section is to assess the role of statistical choices and data quality choices in shaping the result.

survival Cox proportional hazard regression of time to death, with inference based on permutation test of randomization unit

Secondary analyses of mortality.

All secondary analyses will be sharply distinguished from the primary prespecified analysis and will be identified as such. Secondary analyses include outcome variables or planned subsets which contribute either additional insight or address different scientific questions than the primary analysis.

age Poisson regression at the individual level, clustering on community, including age as a covariate (we will both adjust for age, as well as examine age-treatment interactions)

age Negative binomial regression, reporting using the same age categories as in MORDOR Niger/Malawi/Tanzania (1–5 months, 6–11 months, 12–23 months, 24–59 months)

timing Cramer–von Mises comparison of death time conditional on mortality, between arms; comparison of death time density between arms

the number of azithromycin doses received Logistic regression at the individual level, clustering on community, using the number of azithromycin doses as the primary predictor

factorial analysis Logistic regression at the individual level, clustering on community, using the interaction term for the two factors, the community and individual arms

Additional secondary outcomes.

nutritional status Weight, height, and MUAC will be analyzed. MUAC will be compared between arms using clustered linear regression (clustered on community). MUAC will be available in all 341 communities; weight and height will only be available in specific prespecified communities which are located in the HDSS.

malaria Malaria parasitemia is assessed using thick smears. The available data will be the fraction of the sampled children who are positive. This is a longitudinally measured fraction, and we propose modeling this using clustered linear regression on square-root transformed data (with clustered logistic regression as a sensitivity analysis). These data are only available in the 48 HDSS communities.

antibiotic resistance Like malaria parasitemia, phenotypic antibiotic resistance is a binary outcome at the individual level, and a fraction at the community level. The same analytic template as for malaria parasitemia will be used.

microbiome Intestinal and nasopharyngeal microbiome will be assessed. We propose to examine L2-norm (Euclidean) distance using PERMANOVA with permutations at the level of the randomization unit. We will also also examine L1 and diversities (L2 and L1, or Simpson's and Shannon's). This outcome, like malaria and antibiotic resistance, is only available in the 48 communities in the HDSS.

cause of death A verbal autopsy questionnaire for children will be used to collect data for verbal autopsies[**?**]; details are provided in the MOP. Cause of death will be assigned according to an algorithm based on a published verbal autopsy hierarchy. Cause of death will be compared with the use of the chi-square statistic, with clustering taken into account by community-level, using a permutation test to obtain the p-value.

2.2 Interim Monitoring

Efficacy. The trial is designed specifically to collect three years of data, because of

interest in whether the treatment effect is durable after the first. However, in case of a large effect found after one year, it may be desirable to expand treatment to all communities.

A single interim analysis conducted at alpha of 0.001 will be conducted. Specifically, the interim analysis will be conducted when mortality data are available for the first year (two study periods).

The final analysis would be conducted at alpha 0.049.

Futility. An interim analysis of futility is proposed, to be finalized in consultation with the Data and Safety Monitoring Board. Specifically, we propose to conduct negative binomial regression on village-level mortality counts comparing the treatment and control arms (using a common aggregation parameter) using only data from the first year. This analysis will be conducted using simulation. We suggest consideration of the conditional power to detect a 30% effect. If this drops below 20% at the interim analysis, discontinuation of the trial or other changes to the protocol may be made in consultation with the Data and Safety Monitoring Board.

Interim analysis will be executed by the trial biostatistician at the central site.

3 Sample Size Considerations

We use the following formula (cited from [**?**]):

$$c = 1 + \left(z_{\alpha/2} + z_{\beta}\right)^2 \frac{(\lambda_0 + \lambda_1)/y + k^2(\lambda_0^2 + \lambda_1^2)}{(\lambda_0 - \lambda_1)^2}$$

where

c is the number of clusters per arm,

y is the number of person-years per cluster,

λ_0 is the rate in the control group,

λ_1 is the rate in the treatment group,

k is the coefficient of variation in both groups,

α is the alpha level,

β and is the type II error rate.

- The base rate for mortality is assumed to be 0.02 per year in the target age range.
- We assume the same coefficient of variation for village-level mortality as for the Niger site, i.e. 0.34.
- We assume that 16.7% of the population is in the target range of 1–59 months.
- We assume an average community size of 1000 people.
- We assume 10% loss to follow-up per year.
- The effect size is 0.1365.

The number of clusters per arm for Burkina Faso is 170, and the total number of clusters for Burkina Faso is 340.

The sample size is fixed at 341 clusters at the begining of the study.

We estimate that 341 clusters will provide 80% power to detect a 13.65% reduction in mortality in 1-59 month age group.

Power for secondary outcomes

All analyses assume a two-sided alpha of 0.05 and are anticipated to provide a power of at least 80%.

nutritional status The proposed sample size will be sufficient to detect a standardized effect size of 0.108 for weight and height (in the HDSS communities), and of 0.0401 for MUAC (in all communities). We assume an ICC of 0.015.

malaria We assume a placebo parasitemia of 0.2 and an ICC of 0.056. We anticipate at least 80% power to detect a difference of 0.083 between the arms.

antibiotic resistance We assume a placebo drug resistance of 0.05 and an ICC of 0.1. We anticipate at least 80% power to detect a difference of 0.082 between the arms.

4 Randomization

Of the 341 communities available in Nouna district, 48 of these fall within an existing HDSS. We will **stratify** the randomization within this group.

Considering only the 48 HDSS communities, note that an unstratified randomization which allocated each community to **Azithro** with probability 1/2 would yield an approximate 3:2 or worse imbalance (in either direction) with a probability of approximately 8.2%.

The randomization will be conducted using R. The function `sample` with option `replace=FALSE` will be used to conduct the random shuffle. Note that the choice of the random number seed completely determines the randomization. To ensure the integrity of the randomization, we will use the procedure we used for MORDOR/Malawi.

5 Abbreviations

ANCOVA Analysis of covariance

DSMC Data and Safety Monitoring Committee

HAZ height for age Z score

HDSS Health and demographic surveillance system

MUAC Mid upper arm circumference

SAP Statistical Analysis Plan

WAZ weight for age Z score

WHZ weight for height Z score

6 Revision History

5 Oct 2018 Another two pre-specified secondary analyses were added: 1. the number of azithromycin doses received; 2. factorial analysis.

1 Aug 2019 Power calculation and sample size updated.

16 Jan 2020 Changed the title to CHAT.

28 Jan 2020 Took the sensitivity analysis out from Statistical considerations under 2.1.1 Mortality Trial at page 5.

15 June 2022 Changed person time calculation to assign 0 person-time to individuals who are lost to followup or missing during the inter-census interval.

13 Feb 2023 Specified interaction on the additive scale; specified the by-phase subgroup analysis.
